# 

**Published:** 1845-10

**Authors:** 


### INDEX TO VOL. XX

OF THE

### BRITISH AND FOREIGN MEDICAL REVIEW.

| | PAGE |
|---|---|
| Abortion, treatment of . . | 526 |
| Abscess, retro-pharyngeal . | 558 |
| Absorption and ulceration, on . | 291 |
| Acephalocysts, origin of . . | 407 |
| Acidity, predisposing cause of disease | 341 |
| Acids, mineral, as poisons, tests for | 327 |
| Aconite, its action on the system . | 465 |
| its therapeutic action . | 467 |
| poisonous effects of . | 469 |
| Aconitum napellus, history of . | 464 |
| Adams, Mr., translation of P. Ægineta | 206 |
| Addison, on nutrition and inflammation | 121 |
| Ægineta, Paulus, translation of . | 206 |
| Ague and phthisis, antagonists . | 217 |
| Air in the veins, during operation | 87 |
| Air and exercise, effect of on health | 109 |
| Albuminuria, on the cause of . | 107 |
| causes of . . . | 265 |
| pathology and treatment of | 266 |
| Allen, Mr. hist. of York dispensary | 204 |
| Amenorrhea, report on . . | 539 |
| Amentia, M. Pinel on . . | 25 |
| Amputation, on the operation of . | 54 |
| of upper maxillary bone | 195 |
| Anatomical observations by Goodsir | 289 |
| Anatomy and physiology, Dr. Budd on | 509 |
| Aneurism, dissecting, of the aorta . | 94 |
| of the external iliac . | 96 |
| collection of treatises on | 206 |
| on compression in . | 434 |
| Animal chemistry, Simon's treatise on | 206 |
| Animals, captive, consumption in . | 104 |
| Antagonism of disease, phthisis and ague . . . | 217 |
| Arteriotomy, Dr. Pancoast on . | 45-47 |
| Aorta, obliterated . . . | 225 |
| Apoplexia neonatorum . . | 551 |
| Apoplexy, collection of cases of . | 197 |
| statistics of . . . | 198 |
| contraction of limbs in . | 258 |
| Appendix vermiformis, perforation of | 222 |
| Arsenic, history of as a poison . | 329 |
| Artery, humeral, wounds of . | 48 |
| Ascarides, origin of . . . | 417 |
| Asphyxia neonatorum . . | 551 |
| Association, Provincial, transactions of | 508 |
| Asthenopia, Dr. Mackenzie on . | 517 |
| Asylum for insane of middle classes | 479 |
| Atmosphere, its effect on diseases | 102 |
| Atmosphere, how purified . | 344 |
| Atrophia ablactatorum . . | 558 |
| Auscultation of the heart . | 180 |
| cerebral . . | 255 |
| Ascites, observations on . . | 241 |
| Ballingall, Sir G., his military surgery | 202 |
| Bartlett, Dr. on medical science . | 140 |
| Bellingham, Dr., on compression in aneurism . . . | 434 |
| Bennett, Dr. his medical report . | 209 |
| Bethlem hospital, reports of . | 471 |
| great improvements in | 472 |
| Biliary calculi and jaundice . | 244 |
| Bird, Dr. Golding, on urinary deposits | 58 |
| Bleeding, its effects in rheumatism . | 185 |
| Blood, plasticity of in inflammation | 71 |
| pathological analysis of . | 76 |
| condition of in pneumonia | ib. |
| theory of inflammation of . | 77 |
| poisons to be found in . | 320 |
| predisposing causes of disease in . . . | 341 |
| hydatids in . . . | 412 |
| venous, morbid relations of | 430 |
| Bone, on structure and economy of | 305 |
| Bones, on the resection of . | 51 |
| Books received for review . | 288 |
| Bouchut, M. on diseases of infants | 357 |
| Bourgery and Pancoast . . | 39 |
| Bowerbank, on structure of shells | 376 |
| Bowman and Todd, their physiology | 201 |
| Brachet, M. on hypochondriasis . | 364 |
| Brain, a double organ . . | 1 |
| M. Pinel on the pathology of | 18 |
| case of cysticercus of the . | 84 |
| softening of . . | 228 |
| abscess of . . . | ib. |
| hypertrophy of . . | ib. |
| scirrhus of . . . | ib. |
| affections of in heart disease | 262 |
| hydatids in . . | 409 |
| hypertrophy of . . | 555 |
| Bridewell and Bethlem hospitals . | 471 |
| Bronchi, cases of tubular expectoration from . . . | 96 |
| Bronchi, foreign bodies in . | 245 |
| Bronchial glands, disease of . | 252 |
| Bronchitis, epidemic . . . | 245 |
| Bronchitis, Valleix and Dunglison on | 441 |
| its diagnosis from other diseases | 443 |
| Bronchocele, endemic, treatise on | 168 |

PAGE
Budd, Dr. W. his retrospective address 509
Burrows, Dr. his case of carcinoma 90
Cæsarean section . . . 534
Calculous diseases in India . 215
Campbell, Dr. treatise on midwifery 208
Capillary attraction, experiments on 156
Capillaries, of the circulation in . 157
Carcinoma of the lungs, case of . 90
cell-theory of . . 220
Carpenter, Dr. on structure of shells 367
Cartilage of joints, inflammation of 113
Cartilage, process of absorption in 293
Catalepsy of thirteen years' duration 263
Cataract, Mr. Charles Guthrie on . 512
Cells, progressive metamorphoses of 73
regressive metamorphoses of 72
contagious, inoculation of . 213
the element of secretion . 294
Cephalhæmatoma . . . 552
Cerebral auscultation . . 255
diseases, causes of . . 33
Chelius, his manual of surgery . 110
Childhood, diseases of . . 554
Children, treatise on the diseases of 205
physical education of . 357
plurality of . . 529
Chorea, observations on . . 262
Choroid and retina, inflammation of 281
Christison, Dr. on poisons . . 317
Chronic diseases, on the causes of 102
Circulation in the capillaries . 157
Circulatory system in insects . 287
Climate and meteorology, treatises on 215
Climacteric disease . . . 272
Clinical teaching, remarks on . 176
Coagula, organization of . . 88
Colica pictonum, fatal case of . 222
Compression, its use in aneurism . 434
Conjunctiva, inflammation of the . 273
Constipation and ileus . . 242
Constitution, varieties of . 314
Consumption, on the causes of . 102
in different classes . 423
number of deaths from 428
Contagion, observations on . 211
experiments on . 406
Contraction of the limbs in apoplexy 258
Convulsions of infants . . 363
of children . . 555
Cooper, Mr. B. on air in the veins . 87
Cooper's, Mr. fatal ovarian operation 89
Copeman, Mr. his cases of apoplexy 197
Cornea, inflammation of . . 277
ulceration of . . . 279
mortification of . . 279
wounds of . . . 280
Corpuscles, white, and pus-cells, alleged identity of . . . 122
Cowan, Dr. his retrospective address 509
Cretinism, causes of . . . 213
report on . . . 564

PAGE
Crosse, Mr. on inversio uteri . 511
Crystalline lens, inflammation of . 282
Cystine, chemical pathology of . 64
Cysticercus, case of in the brain . 84
Dalrymple, Mr. on spermatozoa . ib.
on coagula . 88
Davy, Dr. on meconium . . 93
Day, Dr. his translation of Simon 206
Delirium, acute, M. Pinel on . 18
chronic, causes of . 22
Delivery, signs of . . . 529
Dementia, simple . . . 24
Dentition, and diarrhea, connexion of 359
Delirium tremens, cases of . . 262
Deposits, urinary, Dr. G. Bird on . 58
Diabetes mellitus, treatment of . 270
Diagnosis, medical, the art of 308-316
Diarrhea and dentition, connexion of 359
of children 361-559
Dictionary, medical, Dr. Palmer's 207
Directory, London medical . 205
Disease, predisposing causes of . 340
analysis of the phenomena of 346
Dislocation of the ulna . . 138
Draper, Dr. on plants . . 153
Duality of the mind, Dr. Wigan on 1
Dunglison, Dr. on therapeutics . 441
human health . 519
Dysentery, epidemic in France . 239
Dysmenorrhœa, report on . . 539
Earthy salts of urine, pathology of 67
Eccentricities, surgical, M. Mayor's 461
Echinococcus, origin of . . 407
Eisenberg, Mr. on the foot . 521
Elephantiasis græcorum . . 267
Eliottson, Dr. on mesmeric diagnosis 287
Empyema, diagnosis of . . 248
in pleurisy . . 451
Endocarditis and pericarditis 188-223-547
Endocardial and exocardial sounds 178
Epilepsy, M. Pinel on . . 31
observations on . . 261
and mania, digitalis in ib.
Epizootic disease in India . 352
Esquirol, M. on mental maladies . 520
Erichsen, Mr. on aneurism . 206
Exeter, on the mortality of . 523
Exhalation, cutaneous, in disease . 312
Exhausting diseases, Sir J. Eyre on 203
Exocardial and endocardial sounds 178
Exploration of disease, internal . 311
Eyelids, on the motion of the . 133
adhesion of to the globe . 134
Eyre, Sir J. on exhausting diseases 203
Falck, M. on bronchocele . 168
Fear, its effects on public health . 215
Fever, geological causes of . 211
hereditary predisposition to ib.
epidemic, peculiar, of Scotland 234
typhoid, remarks on . 235
congestive, of America . ib.

PAGE
Fever erysipelatous . . . 237
intermittent, state of spleen in ib.
treatment of . ib.
theory of . . . 346
state of the blood in . ib.
puerperal . . . 536
Fishes, Prof. Owen on the teeth of 391
Fistula, vesico-vaginal . . 547
Fleming, Dr. his treatise on aconite 464
Fœtus, on the diseases of . . 548
Foot, human, treatise on . . 521
Foramen lacerum, contraction of . 230
Foreign bodies in the bronchi . 245
Fourcault, M. causes of chronic diseases 132
Fractures, Chelius on . . 115
Gall-bladder, enormous distension of 222
Gangrene of the lung . . 226
Gautier, M. on iodine in syphilis 482
Gibert, M. on diseases of the skin . 521
Giles, Mr. his case of malformation 511
Gland, thymus, treatise on . 159
Glands, lymphatic, structure of . 297
Glandular system, predisposing to disease . . . . 344
Glisson's capsule, anatomy of . 135
Goodsir, Messrs. anatomical and pathological observations by . 289
Gout and rheumatism . . 268
Green, Dr. on the seat of tubercle 97
Greenhow, Mr. case of diseased ovary 90
Groin, the, anatomy of . . 139
Guthrie, Mr. on prevention of disease 202
Guthrie, Mr. C. on cataract . 512
Guy, Dr. on the effect of employments on health . . . 421
Gymnastics, its effects on health 109
Hæmatemesis, fatal case of . 239
Hassing, M. on iodine in syphilis . 482
Hawkins, Mr. C. on carcinoma of thyroid . . . . 85
Headache, effect of aconite on . 467
Health, effect of employments on 421
Health, human, Dr. Dunglison on 519
Heart, Dr. Latham on diseases of . 175
on the sounds of . . 177
etiology of diseases of . 212
aneurisms of . . 224
needles in parietes of . ib.
tubercles of . . . 225
abnormal openings in . ib.
diseases of its orifices and valves ib.
on adhesions of . . 253
hypertrophy of in old people 254
valvular disease of . ib.
of digitalis in diseases of 255
diseases of . . . 262
observations on diseases of 453
hypertrophy of . . 458
Hemorrhage, meteorology of . 212
uterine . . 535

PAGE
Hemiplegia, from obstructed circulation 259
Hemiplegia, connected with syphilis ib.
Hereditary predisposition to ague . 211
disease 315
Hernia, radical cure of . . 56
Hesse, endemic bronchocele in . 170
Hey, Mr. his case of aneurism . 96
Hip, anchylosis of, operation for . 50
Hip-joint disease, account of . 114
Hocken, Dr. on inflammation of the retina . . . . 510
Hood, Mr. on the diseases of children 205
Homicidal mania . . . 27
Hooping-cough, suspended by other diseases . . 363
statistics of . ib.
report on . 556
Horn developed in the human skin 87
Humidity . . . . 104
Hunt, Dr. his translation of Esquirol 520
Huschke, M. anatomy of the viscera 419
Hydatids, history of . . 407
produced by inoculation 413
Hydrocephalus in youth . . 260
acute . . 554
Hydrocyanic acid, mode of action of 318
as a poison . 333
Hydrophobia, observations on . 264
Hydro-pneumo-pericardium . 255
Hypertrophy of the brain in children 555
Hypochondriasis, treatises on . 364
pathology of . 365
cerebral origin of ib.
causes of . 369
seat of . . 370
treatment of . 371
Hypothesis and law, relation of . 142
Hysteria, M. Pinel on . . 31
Icterus neonatorum . . . 553
Ileum, perforation of . . 221
recovered from 243
Ileus and constipation . . 242
Incontinence of urine in childhood 559
Induction, its real character . 142
Induration of cellular tissue in infants 553
Infantile mortality . . 549
Infants, treatise on the diseases of 357
peculiarity of diseases of . 550
Infection, purulent, theory of . 218
Inflammation, on plasticity of blood in 71
Mr. Addison on . 121
in different tissues . 273
Influenza, epidemic, account of . 232
history of . . 444
Inoculation, experiments on . 406
of hydatids . . 413
Insane of the middle classes, asylum for 479
Insanity, new views of, by Dr. Wigan 11
Instincts, perversion of . . 25
Intemperance, influence of on health 422

PAGE
Intermittent fever and phthisis, relation of . . . 105
Inversio uteri, Mr. Crosse's essay on 511
Iodide of potassium in syphilis, proper dose of . . . 486
Iodine, on the treatment of syphilis by 482
Iris, inflammation of . . 280
Iritis, effects of mercury on . 191
Jaws, on the diseases of . . 195
Joint, formation of artificial . 49
Jones, Dr. B. on uric acid in the urine 90
Dr. Bence, on oxalate of lime in the urine . . 92
Mr. Wharton, on inflammation of the eye . . 273
Kidney, on the functions of . 125
Kiestein, Dr. Golding Bird on . 69
Labour, management of . 528
preternatural . . 529
premature . . 534
Lactation, observations on . 536
Laryngitis, cases of . . 91
Latham, Dr. on diseases of the heart 175
Lectures, Dr. Latham's clinical . ib.
Liver, on diagnosis of diseases of . 199
tumour connected with . 243
as predisposing to disease . 343
hydatids in . . 411
Livers, diseased, tropical, theory of 343
Lungs, inflammation of, in rheumatism 184
defective expansion of . 213
gangrene of . . 226
cirrhosis of . . . 228
abscess of . . 246
gangrene of . . . 247
carcinoma of . . 253
predisposing causes of disease in 344
Lymphatic system predisposing to disease . . . . . 341
Macilwain, Mr. on tumours . 127
Mackenzie, Dr. on affections of the eye 517
Malaria, its active principle . 209
Male, Dr. death of, from aconite . 469
Malformation, congenital, of the urinary organs . . . 511
Mammary fascia, description of . 135
Man, physiological anatomy of . 201
Mania, acute, M. Pinel on . 18
Mayor, M. his surgical eccentricities 461
Measles, inoculation of . . 211
report on . . 559
Measuring, medical, art of . 311
Mcconium, Dr. Davy, on the composition of . . . . 93
Medical science, philosophy of . 140
Medicine, practical, Dr. Cowan on 509
Medico-Chirurgical Transactions . 83
anatomy, treatise on 133
Meningitis, cerebro-spinal, epidemic 256
Menorrhagia, report on . . 539

PAGE
Menstruation, period of in France . 138
vicarious . . 540
Mental maladies, treatise on . 520
Mercury, its action in inflammation 190
as a remedy in pericarditis 192
as a poison, detection of 332
Meteorology and climate, treatises on 215
Michea, M. on hypochondriasis . 364
Microscopical observations on the blood 121
Midwifery, Dr. Campbell's treatise on 208
Dr. West's report on 525
statistics of . . 536
Miliary fever, account of epidemic 233
Military surgery, outlines of . 202
Mind, Dr. Wigan, on duality of 1
Mollities ossium, on pathology of 100-272
Monomania, acute, M. Pinel on . 21
Mortality of infants . . 549
Mucous membrane, causes of disease from . . . . 341
Muscæ volitantes, Dr. Mackenzie on 517
Muscular system predisposing to disease 345
Myriapoda, natural history of . 487
development of . . 490
Nassau, on endemic bronchocele in 168
Necrosis, M. Petrequin on . 138
Needles in the parietes of the heart 224
Nervous diseases, theory of . 220
system predisposing to disease 345
in insects, Newport on 487
Neuralgia, effect of aconite in . 467
Newport, Mr. researches in physiology 487
Non-restraint in Bethlem . . 476
Nutrition, Mr. Addison on . 121
Odontography, Professor Owen's . 376
Œsophagus, dilatation of . . 221
Omental sacs in hernia, Mr. Hewett on 93
Operations, surgical, on the jaws . 195
Ophthalmia, epidemic, pathology of 82
Opium as a poison, detection of . 332
Organic surgery, Mr. Macilwain on 127
Organization, effect of physical agents on . . . . . 154
O'Shaughnessy, Mr. on the surgery of the jaws . . . . 195
Ottley, Mr. on cysticercus . . 84
Ovaria, diseases of . . . 545
extirpation of . . 546
Ovarian cyst, fatal operation for . 88
tumours, on operations for 101
Ovarium, diseased, removal of . 90
Ovum, a cause of preternatural labour 533
Owen, Professor, his odontography 376
Oxalate of lime, pathology of . 64
in the urine . 92
Paget, Mr. on obstructions in the arteries . . . . 93
Palate, cleft, origin of . . 134
Palmer, Dr. his Pentaglott Dictionary 207
Pancoast, Dr. his operative surgery 39

40

LIBRARY OF THE

Orleans Parish Medical Society

PAGE
Pancreas, diseases of . . 244
Paralysis, general, of mania . 29
treated by galvanism . 260
Paraplegia, treatment of . . 260
Parasitical animals in man . 230
Pathological anatomy of scurvy 147-9
observations by Goodsir 289
Pathology, cerebral, M. Pinel on . 18
of the blood and inflammation 71
general, treatise on . 216
elements of . 339
Patient, methodical examination of 310
Percussion, medical, art of . 311
Perforation of intestinal tube . 221
Pericarditis, Dr. Latham on : 182
diagnosis and treatment of 454
Pericardial sounds, account of . 179
Periodicity of disease . . 216
Peritonitis, strumous . . 240
Petrequin, M. on medico-chirurgical anatomy . . . 133
Phillips, Mr. on ovarian operations 101
Philosophy of medical science, Dr. Bartlett's . . . 140
Phlebitis, intra-cranial . . 229
Phthisis in captive animals . 104
influence of employment on 214
and ague, antagonists . 217
statistics of . . 250
treatment of . . ib.
from irritating substances 251
diagnosis and treatment of 447
of children . . 558
Physiognomy, state of in diseases . 313
Physiological anatomy and physiology of man . . . . 201
Physiology of the thymus gland . 159
Pinel, M. S. on cerebral pathology 18
Placenta, human, structure of . 299
Plants, on the organization of . 153
Pleurisy, diagnosis of . . 247
Valleix and Dunglison on 450
Pneumonia, action of lead on . 81
Dr. Addison on . 226
Pneumo-thorax, history of . . 249
Pneumonia treated, without bleeding 252
in infants, account of . 362
of children . . 556
Poisoning, on the evidences of . 322
Poisons, Dr. Christison's treatise on 317
physiological action of . ib.
on the action of . . 321
on antidotes to . . 323
Polypus, true, of the auricle . . 223
Porrigo, of vegetable origin . . 267
Pregnancy, on the signs of . . 525
disorders of . . 526
extra-uterine . . ib.
fallopian, cases of . 527

PAGE
Pressure, cure of aneurism by . 434
its advantages 438
Prevention of phthisis . . 108
Prolapsus uteri, mistaken . . 128
Provincial Association, transactions of 508
Pseudo-plastic processes, Dr. Zimmermann on . . . 71
Publications in parts, animadversions on . . . 120
Puchelt, M. on the venous system 430
Puerperal fever . . . 536
Pulmonary artery, on obstructions in 93
Purpura, fatal case of . . 270
Purpurine, chemical pathology of 64
Pyrosis, case of . . . 239
Ranula, M. Petrequin on . . 134
Report on the progress of medicine 209
Reproduction of lost parts . . 307
Mr. Newport on 487
Reptiles, Professor Owen on the teeth of . . . . 398
Respiration in infants, state of . 358
Retina, inflammation of . . 281
Dr. Hocken on 510
Rheumatism and pericarditis, relation of . . . . 183
Rheumatism, acute, treatment of . 184-6
transmission of, to the fœtus (?) . . 215
effect of aconite on . 467
Ribs, on the motion of . . 135
Rickets in children . . . 564
Rings, inguinal and femoral, action of 139
Russia, of scurvy in . . 147
Salts which dissolve fibrine . . 75
Samson-Himmelstiern, Dr. on scurvy 147
Scarlatina, report on . . 560
Schultz, M., his elements of pathology 339
Sciatica, remarks on treatment of . 265
Science, definition of . . 141
Sclerotica, inflammation of . . 276
Scotland, peculiar epidemic fever in 234
Scrofula in childhood . . 564
Scurvy, marine, treatise on . . 147
causes of . . . 148
morbid anatomy of . . 149
epidemic . . . 271
Secretion and secreting structures, observations on . . . 293
Seminal fluid contained in a cyst . 100
Shapter, Dr. on the mortality of Exeter 523
Shells, on the structure of . 376
Sheppard, his translation of Gibert . 521
Siebert, M. on medical diagnosis . 308
Silver, oxide of, its therapeutic value 203
Simon, Mr. on the thymus gland . 159
Skin, experiments on the functions of 106
diseases of . . . 266
M. Gibert on diseases of . 521

| | PAGE |
|---|---|
| Smallpox in Calcutta, report on | 345 |
| Soemmering, anatomy of the viscera | 419 |
| Solly, Mr. on molities ossium | 100 |
| South, Mr. his translation of Chelius | 110 |
| Spermatozoa in hydrocele, cause of | 84 |
| Spleen, diseases of | 222 |
| removal of | 244 |
| Stammering, on the cure of | 135 |
| Stanley, Mr. on ruptured ureter | 83 |
| Stewart, Dr. on smallpox in Calcutta | 345 |
| Stomach, perforation of | 221 |
| diseases of | 238 |
| Sunbeams, action of, on plants | 155 |
| Surgery, operative, Dr. Pancoast's | 39 |
| Chelius's manual of | 110 |
| Surgical operations too lightly undertaken | 111 |
| Suicide, M. Pinel on | 26 |
| Sydenham Society, works published by | 206 |
| Symphyseotomy | 534 |
| Syncope, alarming, from air in the veins | 87 |
| Synovial membrane, inflammation of | 113 |
| Syphilis, on its treatment by iodine | 482 |
| Teeth, on the structure of | 378 |
| elements composing the | 378-383 |
| development of | 383 |
| Tetanus, case of | 264 |
| Testicles, descent of | 136 |
| Testis, on its structure and secretion | 296 |
| Tetanus, Chelius on | 115 |
| Therapeutics, infantile | 551 |
| Thoracic inflammation, pathology of | 79 |
| bloodletting in | 80 |
| nitre in | 81 |
| tartar emetic in | 81 |
| Thymus gland, treatise on | 159 |
| Thyroid gland, carcinoma of | 85 |
| Tic douloureux, observations on | 264 |
| Todd, Dr. case of dissecting aneurism | 94 |
| Todd and Bowman, their physiology | 201 |
| Toothache, effect of aconite on | 467 |
| Trichina spiralis | 415 |
| Trismus neonatorum | 552 |
| Tubercle, on the seat of | 97 |
| pathology of | 218 |
| in the bones | 220 |
| Tumour in the hypochondrium, case of | 99 |
| Tumours, Mr. Macilwain on | 127 |
| treatment of | 128 |
| Tympanitis, case of | 243 |
| Ulceration and absorption, on | 291 |
| Ulna, dislocation of | 138 |
| Ureter, cases of ruptured | 83 |
| Urethra, on the length of | 136 |
| Uric acid and its combinations | 62 |
| acid diathesis, treatment of | 63 |
| acid, its state in the urine | 90 |
| Urinary deposits, Dr. Golding Bird on | 58 |
| Urinary organs, case of malformation | 511 |
| Urine, chemical physiology of | 60 |
| properties of | 60 |
| oxalic, Dr. Bird on | 65 |
| on coagulable | 75 |
| on oxalate of lime in | 92 |
| Uterine hemorrhage | 535 |
| Uterus, inversion of, Mr. Crosse on | 511 |
| Uterus, rupture of | 526 |
| investigation of disease of | 540 |
| inversion of | 530 |
| rupture of | 531 |
| displacement of | 540 |
| retroversion of | 541 |
| inversion of | ib. |
| cervix, inflammation of | 542 |
| polypus of | 543 |
| fibrous tumours of | 544 |
| malignant diseases of | ib. |
| Vaccination in India, difficulties of | 345-353 |
| and variola, report on | 560 |
| Valleix, M., guide to medical practice | 441 |
| Variola and vaccination, report on | 560 |
| Vascular system predisposing to disease | 343 |
| Veins, operations on | 46 |
| Venereal disease treated by iodine | 482 |
| Venosity or venous diatheses | 431 |
| Venous system, pathological relations of | 430 |
| Vena azygos, fatal rupture of | 225 |
| Vesico-vaginal fistula | 547 |
| Vernois, M. on liver diseases | 199 |
| Vision, Dr. Mackenzie on | 517 |
| Viscera and organs of sense, anatomy of | 419 |
| Vitreous humour, inflammation of | 283 |
| West, Dr. his report on midwifery | 525 |
| Wigan, Dr. on duality of mind | 1 |
| Wilson, Mr. Erasmus, his case of horn in the skin | 87 |
| Wilson, Dr. on laryngitis | 91 |
| Worms, intestinal, origin of | 416 |
| Wounds of the head, Chelius on | 115 |
| York dispensary, history of | 204 |
| Zimmermann, Dr. J. on pseudo-plastic processes | 71 |

END OF VOL. XX.

C. AND J. ADLARD, PRINTERS, BARTHOLOMEW CLOSE.

